# Spirituelle Kompetenzen von Gesundheitspersonal in der Notfall- und Intensivversorgung – eine prospektive Fragebogenstudie

**DOI:** 10.1007/s00063-024-01185-1

**Published:** 2024-10-23

**Authors:** J. Schwartz, T. Tenge, K. Lanhenke, S. Meier, M. Schallenburger, Y.-N. Batzler, T. Roser, D. Wetzchewald, M. Neukirchen

**Affiliations:** 1 Interdisziplinäres Zentrum für Palliativmedizin, Medizinische Fakultät und Universitätsklinikum Düsseldorf, Heinrich-Heine-Universität Düsseldorf, Düsseldorf, Deutschland; 2 Klinik für Anästhesiologie, Medizinische Fakultät und Universitätsklinikum Düsseldorf, Heinrich-Heine-Universität Düsseldorf, Düsseldorf, Deutschland; 3 Seminar für Praktische Theologie, Universität Münster, Münster, Deutschland; 4 Institut für Notfallmedizin, Arnsberg, Deutschland

**Keywords:** Religiosität, Kritisch Erkrankte, Palliativmedizin, SCCQ, Schulungsbedarf, Religiosity, Critical illness, Palliative care, SCCQ, Need for training

## Abstract

**Hintergrund:**

In der Intensiv- und Notfallversorgung werden Patient:innen und ihre Zugehörigen mit möglicherweise existenziellen Krisen konfrontiert. Hier kann Spiritual Care eine zusätzliche Versorgungsressource sein. Für das Erkennen und Adressieren dieser Bedürfnisse sind spirituelle Kompetenzen des Gesundheitspersonals notwendig.

**Ziel der Arbeit (Fragestellung):**

Wie sind die spirituellen Kompetenzen von Gesundheitspersonal der Intensiv- und Notfallversorgung ausgeprägt? Gibt es Unterschiede zwischen den Professionen und Geschlechtern? Was sind Einflussfaktoren für spirituelle Kompetenzen?

**Material und Methoden:**

In der prospektiven Fragebogenstudie wurden Ärzt:innen *aus multizentrischen Kliniken*, die an den Kursen für Intensiv- und Notfallmedizin in *Arnsberg* teilnahmen, sowie Pflegefachkräfte der Intensiv- und Notfallversorgung am Standort* Düsseldorf* eingeschlossen. Mittels Selbsteinschätzung im Spiritual Care Competence Questionnaire (SCCQ) wurden spirituelle Kompetenzen in den folgenden Bereichen erfasst: Wahrnehmungskompetenz, Teamspirit, Dokumentationskompetenz, Selbsterfahrung und proaktive Öffnung, Wissen über andere Religionen, Gesprächsführungskompetenz und proaktive Empowerment-Kompetenz.

**Ergebnisse:**

Von den Befragten waren 465 Ärzt:innen (50 % weiblich, Berufsjahre: MW = 4,0; SA = 3,5) und 86 Pflegefachkräfte (80 % weiblich, Berufsjahre: MW = 12,7; SA = 10,7). Die durchschnittliche SCC betrug im Mittel 2,3 (SA = 0,4) von maximal 4 Punkten, wobei spirituelle und gläubige Befragte eine höhere spirituelle Kompetenz aufwiesen. Unterschiede in spezifischen Kompetenzen zeigten sich zwischen den Professionen und Geschlechtern. Frauen gaben eine höhere Kompetenz im Bereich Wahrnehmung und Gesprächsführung an, Ärzt:innen in der Dokumentationskompetenz.

**Diskussion:**

Insgesamt wird ein Schulungsbedarf bei Gesundheitspersonal im Bereich der Intensiv- und Notfallmedizin deutlich.

**Zusatzmaterial online:**

Zusätzliche Informationen sind in der Online-Version dieses Artikels (10.1007/s00063-024-01185-1) enthalten.

## Abstract (en)

**Background:**

In intensive and emergency care, patients and their relatives are confronted with potentially existential crises. Spiritual care can be an additional resource to address related psychosocial and physical symptoms and to support patients and their relatives. Accordingly, healthcare workers need spiritual skills to recognize and respond to these needs.

**Objectives:**

What spiritual competencies do healthcare workers in intensive and emergency care have? Are there differences between professions and genders? What factors influence spiritual competencies?

**Materials and methods:**

The prospective questionnaire study included physicians participating in intensive care and emergency medicine courses and nurses who were training or working in intensive and emergency care. Self-reported spiritual competencies were assessed using the Spiritual Care Competence Questionnaire (SCCQ), which captures the following areas: perceptual competence, team-spirit, documentation competence, self-awareness and proactive opening, knowledge about other religions, competence in conversation techniques and proactive empowerment-competence.

**Results:**

We included 465 physicians (50% female, years in profession: mean = 4.0, standard deviation [SD] = 3.5) and 86 nurses (80% female, years in profession: mean = 12.7, SD = 10.7). The average SCC was 2.3 (SD 0.4) out of a maximum of 4 points, with higher spiritual competences among spiritual and religious respondents. There were differences in specific competencies between the professions and genders. Women indicated a higher level of competence in the area of perception and conversation skills, physicians in documentation skills.

**Conclusions:**

Overall, there is a clear need to train healthcare staff in the field of intensive care and emergency medicine.

## Einleitung

Konfrontiert mit einem möglichen Versterben erleben schwerstkranke Patient:innen und ihre Zugehörigen auf Intensivstationen und in Notaufnahmen eine existenzielle Bedrohung. Sie erfahren Leiden auf körperlicher, psychischer, sozialer und spiritueller Ebene. Um die spirituellen Bedürfnisse zu adressieren, müssen diese durch das Personal erkannt werden. Hierzu sind grundlegende spirituelle Kompetenzen erforderlich. In dieser Studie wurden spirituelle Kompetenzen anhand des Spiritual Care Competence Questionnaire (SCCQ) bei ärztlichem und pflegerischem Notfall- und Intensivpersonal erhoben.

## Hintergrund und Fragestellung

Spiritualität ist ein Begriff, der heterogen definiert und häufig mit Religiosität in Verbindung gebracht wird. Seit der WHO-Definition von Palliative Care, in der Symptomlinderung nicht nur im Hinblick auf physische, psychische und soziale Aspekte adressiert wird, sondern auch auf spiritueller Ebene beschrieben wird [30], haben spirituelle Bedürfnisse Beachtung in der Versorgung schwerkranker Patient:innen und ihrer Zugehörigen gefunden [12]. In einer deutschen Übersetzung der Definition von Spiritualität der Europäischen Fachgesellschaft für Palliativmedizin wird das multidimensionale Konzept wie folgt beschrieben: „Spiritualität ist die dynamische Dimension menschlichen Lebens, die sich auf die Art und Weise bezieht, in der Personen (Individuen und Gemeinschaften) Sinn, Bedeutung und Transzendenz erfahren, ausdrücken und/oder suchen, und die Art und Weise, wie sie sich mit der Natur, dem Bedeutsamen und/oder dem Heiligen verbinden. Spiritual Care bezeichnet dabei die Sorge um spirituelle Themen, Nöte, Fragen und Ressourcen. Das spirituelle Feld ist multidimensional:

1. existenzielle Herausforderungen (z. B. Fragen nach Identität, Sinn, Leiden und Tod, Schuld und Scham, Versöhnung und Vergebung, Freiheit und Verantwortung, Hoffnung und Verzweiflung, Liebe und Freude);
2. wertorientierte Überzeugungen und Haltungen (individuelle Priorisierung hinsichtlich Beziehung zu sich selbst, Familie, Freunden, Arbeit, dinglicher Natur, Kunst und Kultur, Ethik und Moral und zum Leben an sich);
3. religiöse Überzeugungen und Fundamente (Glaube, Glaubensinhalte und Praktiken, die Beziehung zu Gott oder dem Endgültigen)“ [16, 24].

Für das Adressieren spiritueller Bedürfnisse müssen diese erkannt werden. Hierfür sind grundsätzliche spirituelle Kompetenzen beim Gesundheitspersonal notwendig. Um diese zu erheben, einen Schulungsbedarf zu erfahren und mögliche Trainings zu evaluieren, haben Frick et al. den deutschsprachigen Spiritual Care Competence Questionnaire (SCCQ) entwickelt [13]. Speziell Patient:innen in der Intensiv- und Notfallversorgung sowie ihre Zugehörigen erleben eine Ausnahmesituation, bei der sie mit akuten (Gesundheits‑)Krisen, Unsicherheit in Bezug auf die Zukunft und einer möglichen Lebenslimitierung konfrontiert sein können. Eine Notfall- und Intensivversorgung hat zunächst Stabilisierung und Lebensrettung zum Ziel, die aber nicht in allen Fällen erreicht werden kann. Außerdem ist nicht immer klar, inwiefern sich die Prognose ändert. Daher können sich in solch einer Situation Bedürfnisse entwickeln, die berücksichtigt werden sollten, um der Krise standhalten und Ressourcen finden zu können. Viele Patient:innen und Zugehörige nutzen in solchen Situationen Spiritualität und Religiosität als Copingstrategie [20]. In verschiedenen Studien konnte gezeigt werden, dass das Eingehen auf spirituelle Bedürfnisse den Betroffenen Erleichterung verschaffen kann: Es kann die Lebensqualität verbessern, die Zufriedenheit mit der medizinischen Versorgung steigern und negative psychologische Folgen lindern oder vorbeugen [19, 29]. Auch Gesundheitspersonal hat Spiritualität als relevanten Einflussfaktor erkannt. So wurde vom American College of Critical Care Medicine bereits 2007 eine Leitlinie zur spirituellen Begleitung von Intensivpatient:innen erarbeitet [9]. Spirituelle und religiöse Bedürfnisse zu berücksichtigen, ist außerdem als Teil einer ganzheitlichen pflegerischen Versorgung schwerstkranker Patient:innen identifiziert [3]. Als Hürden zur Erhebung einer spirituellen Anamnese im klinischen Alltag wurden Zeitmangel, ein Fokus auf eher medizinische Inhalte und Unwohlsein beim Ansprechen von Patient:innen mit unterschiedlichen religiösen Ansichten angegeben [1].

In dieser Arbeit wurden die spirituellen Kompetenzen von ärztlichen und pflegerischen Notfall- und Intensivpersonal anhand des SCCQ erhoben. Unterschiede in den Professionen und Geschlechtern sowie Einflüsse auf spirituelle Kompetenzen wurden untersucht. Aus den Ergebnissen kann unter anderem der Bedarf an möglichen Schulungen identifiziert werden.

## Studiendesign und Untersuchungsmethoden

Es erfolgte eine prospektive Fragebogenstudie nach positivem Ethikvotum der Ethikkommission der Heinrich-Heine-Universität Düsseldorf (Studien-Nr.: 2023-2431). Die Prinzipien der Deklaration von Helsinki wurden befolgt. Eingeschlossen wurden Pflegefachkräfte und Ärzt:innen aus der Notfall- und Intensivversorgung. Das ärztliche Kollektiv wurde in den Kursen für Intensiv- und Notfallmedizin der Arbeitsgemeinschaft Intensivmedizin e. V. rekrutiert. Die Befragung der Pflegefachkräfte erfolgte im Rahmen der Fachweiterbildung für Intensiv- und Anästhesiepflege des Bildungszentrums des Universitätsklinikums Düsseldorf sowie beim Personal der Intensivstationen und der zentralen Notaufnahme des Universitätsklinikums Düsseldorf. Die Rekrutierung der Teilnehmer:innen erfolgte 2023 durch eine direkte Kontaktaufnahme über Gatekeeper im Sinne von Kursleitungen und via E‑Mail-Anfrage. Die Teilnahme war freiwillig und die Datenerhebung erfolgte digital mittels der Onlineplattform Unipark (Tivian XI GmbH, Köln, Deutschland). Zu Beginn der Fragebogenbeantwortung erfolgte die Aufklärung zur Studienteilnahme mit Verweisen auf das vorliegende Datenschutzkonzept und die jederzeit bestehende Möglichkeit zum Abbruch der Teilnahme. Die weitere Teilnahme an der Fragebogenbeantwortung wurde dann als informierte Einwilligung gewertet. Erfasst wurden neben soziodemografischen Angaben und Angaben zur Profession, zur Berufstätigkeit, eigenen Spiritualität und Gläubigkeit als individuelle Ausprägung von Religiosität die spirituellen Kompetenzen mittels SCCQ. Der von Frick et al. entwickelte SCCQ besteht aus 26 Fragen und definiert 7 Faktoren:

1. Wahrnehmungskompetenz: spirituelle Bedürfnisse von Patient:innen und Zugehörigen wahrnehmen;
2. Teamspirit: Umgang mit und Austausch über Spiritualität im Team;
3. Dokumentationskompetenz: Kenntnis von Instrumenten zur Erfassung spiritueller Bedürfnisse, Fähigkeit zur nachvollziehbaren Dokumentation;
4. Selbsterfahrung und proaktive Öffnung: Vertiefung eigener Spiritualität, Ansprechen und Raum schaffen für spirituelle Bedürfnisse der Patient:innen;
5. Wissen über andere Religionen und deren Berücksichtigung;
6. Gesprächsführungskompetenz: Fähigkeit, ein offenes Gespräch über existenzielle oder religiöse Themen zu führen;
7. Proaktive Empowerment-Kompetenz: berücksichtigen und bestärken der Spiritualität der Patient:innen.

Die spirituellen Kompetenzen werden dabei wie in der Originalarbeit der Fragebogenentwicklung mittels 4‑stufiger Likert-Skala als Selbsteinschätzung von 1 (stimmt nicht), 2 (stimmt kaum), 3 (stimmt eher) bis 4 (stimmt genau) erfasst. Die Auswertung erfolgte mittels deskriptiver Statistik und Dependenzanalysen (Mann-Whitney-Test, Korrelation nach Spearman-Rho) mittels SPSS (Version 26.0, IBM Corp., Armonk, USA). Die spirituelle Kompetenz insgesamt (Gesamtscore) wurde als Mittelwert aus den 7 Dimensionen der spirituellen Kompetenzen errechnet. Die Daten werden als Mittelwert (MW) und Standardabweichung (SA) oder Häufigkeit und Prozent angegeben.

## Ergebnisse

### Studienpopulation

Insgesamt konnten 465 Ärzt:innen (50 % Frauen; Berufsjahre: MW: 4,0; SA: 3,5; Median 3) sowie 86 Pflegefachkräfte eingeschlossen werden (80 % Frauen; Berufsjahre: MW: 12,7; SA: 10,7; Median 8). Bei den Ärzt:innen kam der größte Anteil aus dem Fachbereich der Inneren Medizin, bei den Pflegefachkräften aus dem Fachbereich Chirurgie. Während die berufliche Zufriedenheit in beiden Gruppen vergleichbar war, war die Berufserfahrung in Jahren im pflegerischen, die Wochenarbeitszeit im ärztlichen Kollektiv größer. Von den 551 Teilnehmer:innen bezeichneten sich 41 % als spirituell, 47 % gaben an, gläubig zu sein. Weitere soziodemografische und berufsbezogene Daten finden sich in Tab. 1.

**Tab. 1:** Demografische Angaben

	Gesamt	Ärzt:innen	Pflegefachkräfte
*Anzahl gesamt (n)*	551	465	86
*Geschlecht (%)*			
Männlich	43,9	48,4	19,8
Weiblich	55,2	50,5	80,2
Fehlende Daten	0,9	1,1	0
*Altersgruppen (%)*			
18–29 Jahre	38,1	37,4	41,9
30–39 Jahre	52,3	56,1	31,4
40–49 Jahre	7,1	6,2	11,6
50–59 Jahre	1,8	0,2	11,6
> 60 Jahre	0,7	0	3,5
*Familienstand (%)*			
Verheiratet	34,5	34,4	34,9
Mit Partner	30,7	31,0	29,1
Geschieden	0,9	1,1	0
Alleinstehend	33,9	33,5	36,0
Verwitwet	0	0	0
*Fachbereich (%)*			
Innere Medizin	54,8	59,8	27,9
Chirurgie/Orthopädie	16,0	11,4	40,7
Anästhesie	19,1	20,9	9,3
Neurologie	3,3	3,9	0
Andere	6,5	3,9	20,9
Fehlende Daten	0,2	0,2	1,2
*Berufserfahrung in Jahren (MW, SA)*	5,4 ± 6,2	3,9 ± 3,5	12,7 ± 10,7
*Durchschnittliche Wochenarbeitszeit in Stunden (MW, SA)*	45,5 ± 9,6	47,5 ± 8,6	34,89 ± 7,3
*Berufliche Zufriedenheit (MW, SA)*			
1 (sehr unzufrieden) bis 5 (sehr zufrieden)	3,6 ± 0,8	3,6 ± 0,9	3,6 ± 0,7
*Spiritualität (%)*			
Ja	41,2	40,4	45,3
Nein	57,4	57,8	54,7
Fehlende Daten	1,4	1,7	0
*Gläubigkeit (%)*			
Ja, unbedingt	19,8	21,1	12,8
Ja, etwas	26,9	28,4	18,6
Eher nein	24,3	23,2	30,2
Nein, gar nicht	28,3	26,5	38,4
Fehlende Daten	0,7	0,9	0
*Religionszugehörigkeit (%)*			
Katholisch	25,8	26,9	24,4
Protestantisch	18,9	18,1	23,3
Muslimisch	17,2	19,8	3,5
Jüdisch	0,2	0,2	0
Andere	8,5	8,8	7,0
Keine	28,3	26,0	40,7
Fehlende Daten	1,1	1	1,2

### Spirituelle Kompetenz

**Abb. 1:**
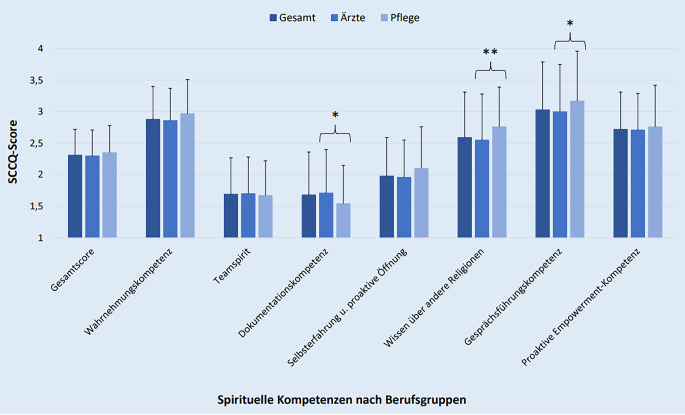
Spirituelle Care Competence – Mittelwertvergleich zwischen ärztlichem und pflegerischem Kollektiv (* *p* < 0,05, ** *p* < 0,01)

Insgesamt war die spirituelle Kompetenz im Gesamtkollektiv im Mittel 2,31 (SA: 0,41), im ärztlichen Kollektiv 2,29 (SA: 0,41) und im pflegerischen Kollektiv 2,35 (SA: 0,42). Im Hinblick auf die spezifischen Kompetenzen (siehe Abb. 1) gaben Ärzt:innen eine höhere Dokumentationskompetenz (MW: 1,71) als Pflegefachkräfte (MW: 1,54) an (U-Wert [U] = 17337, *p* = 0,046, Cohen’s d Effektstärkenwert [d] = 0,25). Pflegefachkräfte berichteten hingegen ein größeres Wissen über andere Religionen (MW: 2,76) als Ärzt:innen (MW: 2,56; U = 16485, *p* = 0,008, d = 0,31) sowie eine höhere Gesprächsführungskompetenz (MW: 3,17) als Ärzt:innen (MW: 3,0; U = 17205, *p* = 0,034, d = 0,22) an. Im Vergleich der Geschlechter zeigte sich, dass Frauen eine höhere Wahrnehmungskompetenz (U = 30814, *p* = 0,001, d = 0,31) sowie Gesprächsführungskompetenz angaben (U = 31629 *p* = 0,004, d = 0,28).

### Einflussfaktoren

**Abb. 2:**
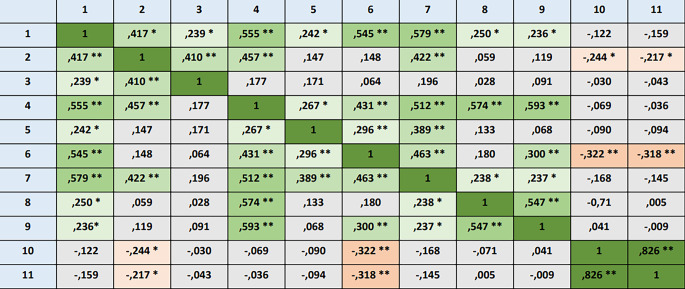
Korrelationsmatrix zur Darstellung der Korrelationskoeffizienten von Zusammenhängen zwischen verschiedenen Faktoren und spirituellen Kompetenzen im pflegerischen Kollektiv, (* *p* < 0,05, ** *p* < 0,01, jeweils 2‑zeitig). *1* Wahrnehmungskompetenz, *2* Teamspirit, *3* Dokumentationskompetenz, *4* Selbsterfahrung und proaktives Öffnen, *5* Wissen über andere Religionen, *6* Gesprächsführungskompetenz, *7* proaktive Empowerment-Kompetenz, *8* Gläubigkeit, *9* Spiritualität, *10* Alter, *11* Berufsjahre

Korrelationen zwischen dem Alter der Teilnehmer:innen, der Berufserfahrung, beruflichen Zufriedenheit, Angaben zu Gläubigkeit und Spiritualität und den spezifischen spirituellen Kompetenzen wurden untersucht, um Zusammenhänge zu ermitteln. Bezogen auf die Gesamtpopulation zeigte sich eine mittlere Korrelation zwischen Gläubigkeit und Spiritualität in Bezug auf die Kompetenz Selbsterfahrung und proaktives Öffnen (Korrelationskoeffizient [r] = 0,461, *p* > 0,001 bzw. r = 0,476, *p* > 0,001). Auch im ärztlichen Kollektiv zeigte sich ein mittlerer Zusammenhang zwischen Gläubigkeit sowie Spiritualität und der Kompetenz Selbsterfahrung und proaktives Öffnen (r = 0,454 bzw. r = 0,446, *p* < 0,001). Es besteht eine geringe Korrelation zwischen beruflicher Zufriedenheit und Teamspirit (r = 0,238, *p* < 0,001). Im pflegerischen Kollektiv zeigte sich ein hoher Zusammenhang zwischen Gläubigkeit und Spiritualität und der spirituellen Kompetenz Selbsterfahrung und proaktives Öffnen (r = 0,454 bzw. r = 0,446, *p* < 0,001). Auch zeigten sich in diesem Kollektiv eine negative mittlere Korrelation zwischen Alter sowie Berufsjahren und der Gesprächsführungskompetenz sowie weitere geringe Korrelationen (siehe Abb. 2). Im Gesamtkollektiv konnten im Hinblick auf Geschlecht und Profession bezogen auf die spirituellen Kompetenzen nur schwache korrelierende Unterschiede verdeutlicht werden.

## Diskussion

Die vorliegende Studie zeigt, dass das Gesundheitspersonal im Bereich der Notfall- und Intensivversorgung die eigenen spirituellen Kompetenzen insgesamt als eher niedrig einschätzt. Dabei wurden besonders die Kompetenzen im Bereich Dokumentation und Teamspirit schlecht bewertet. Zu beachten gilt, dass es sich in dieser Erhebung ausschließlich um Selbsteinschätzungen der befragten Personen handelt. In einer Erhebung beim Gesundheitspersonal in der Psychiatrie und Psychotherapie schätzte das Personal die eigenen Kompetenzen ebenfalls eher als gering ein [14]. In diesem Kollektiv wurde die Dokumentationskompetenz am schlechtesten, die Gesprächsführungskompetenz am besten eingeschätzt. Anders als in unserem Kollektiv war hier die Gesprächsführungskompetenz unter Ärtz:innen signifikant höher als unter Pflegenden. Dabei ist zu beachten, dass teilnehmende Ärtz:innen in unserem Kollektiv mit im Median 3 Berufsjahren weniger Berufserfahrung aufwiesen als Pflegende mit im Median 8 Berufsjahren. Insgesamt war die Berufserfahrung in Jahren in unserem Kollektiv (MW = 5,4) deutlich niedriger als in der Erhebung beim Gesundheitspersonal in der Psychiatrie und Psychotherapie (MW = 19).

In der vorliegenden Erhebung zeigten sich Unterschiede in den spezifischen Kompetenzen zwischen Professionen und Geschlechtern. Bezogen auf den maximalen Punktwert war die angegebene spirituelle Kompetenz der Pflegefachkräfte 58 %, die der Ärzt:innen 57 %. Während bei Pflegefachkräften die Gesprächsführungskompetenz und das Wissen über andere Religion stärker vertreten waren, war es im ärztlichen Kollektiv die Dokumentationskompetenz. Frauen gaben höhere Wahrnehmungs- und Gesprächsführungskompetenzen an. Als Einflüsse ließen sich das jeweils angegebene Ausmaß an Gläubigkeit und Spiritualität besonders im Hinblick auf Selbsterfahrung und proaktives Öffnen ausmachen. Dieser Zusammenhang war in der Erhebung spiritueller Kompetenzen unter Gesundheitspersonal in Psychiatrie und Psychotherapie sogar noch deutlicher ausgeprägt [14]. Die MW der 7 Kompetenzen lassen sich auch mit der Validierungskohorte des SCCQ mit 714 Teilnehmer:innen unterschiedlicher Professionen vergleichen. Hier wurde ebenfalls die Dokumentationskompetenz am schlechtesten, die Gesprächsführungskompetenz am besten bewertet. Die Zusammensetzung der Stichproben aller 3 Studien ist allerdings sehr unterschiedlich. Während in der Validierungskohorte 74 % Frauen und in der psychiatrischen bzw. psychotherapeutischen Erhebung 73 % Frauen waren, waren in unserer Studie nur rund 55 % weiblich. Auch unterschieden sich Alter, Professionen und Fachbereiche zwischen den Kohorten. Als Einfluss auf spirituelle Kompetenzen zeigte das Alter positive Korrelationen sowohl in der Validierungsstudie als auch in der Erhebung bei psychiatrischem und psychotherapeutischem Personal, die in der Validierungsstudie als individuelle Entwicklungsprozesse gewertet wurden [13]. Diese Korrelation konnte in unserem Kollektiv nicht erhoben werden. Allerdings waren Befragte in unserem Kollektiv im Mittel der Altersstufe 30–39 Jahre zugeordnet und damit jünger als in der Validierungsstudie (MW = 41,5 Jahre) oder in der Erhebung beim psychiatrischen bzw. psychotherapeutischen Gesundheitspersonal (MW = 43,2 Jahre).

In Situationen kritischer Erkrankungen sind Zugehörige gefährdet, eine posttraumatische Belastungsreaktion zu entwickeln [2]. In der Begleitung der Patient:innen und auch der Zugehörigen sind kommunikative Kompetenzen des Personals und ausreichende Gesprächsangebote unerlässlich [21]. Andernfalls besteht die Gefahr, dass Zugehörige bei unzureichend empfundener Kommunikation eine komplizierte Trauerreaktion nach Versterben der Patient:innen zeigen [17]. Gelingende Kommunikation ermöglicht auch das Erheben spiritueller Bedürfnisse [18]. Um die spirituellen Bedürfnisse zu adressieren, sind grundlegende spirituelle Kompetenzen erforderlich. Mittlerweile wird die Bedeutung von Spiritualität in der medizinischen Behandlung auf Intensivstationen und in Notaufnahmen zunehmend wahrgenommen [11, 29]. Spiritual Care ist dabei nicht alleinig Aufgabe von Seelsorgenden, sondern ebenso in der Verantwortung des Gesundheitspersonals [4]. Es gilt professionsübergreifend für Pflegende wie Ärzt:innen gleichermaßen, diese Anforderung wahrzunehmen und gleichzeitig die Notwendigkeit für die Verbesserung ihrer Kompetenzen zu erkennen [29]. Mayr et al. wiesen auf die Kluft zwischen selbst eingeschätzter und tatsächlicher Kompetenz in spiritueller Anamnese und die Bedeutung von Fortbildung hin [23]. In einer Befragung von Allgemeinmediziner:innen zeigte sich eine deutliche Diskrepanz zwischen Wunsch und gefühlter Kompetenz [15]. Von Patient:innenseite wurde spirituelle Kompetenz als Ergänzung für die Arzt-Patienten-Beziehung ausdrücklich gewünscht [5] und z. B. von der Deutschen Gesellschaft für Psychiatrie und Psychotherapie in einem Positionspapier für diese Behandlungskontexte gefordert [27].

Um spirituelle Bedürfnisse von Patient:innen und Zugehörigen als Teil eines großen Aufgabenkomplexes einer multidimensionalen (End-of-life‑)Versorgung der Intensiv- und Notfallmedizin zu identifizieren, gibt es eine Reihe verschiedener Erfassungsinstrumente [7, 22, 26, 28], die z. T. unterschiedliche Patientenkollektive adressieren. Der Spiritual Needs Questionnaire (SpNQ) ermöglicht dabei die Beurteilung spiritueller Bedürfnisse von religiösen und nichtreligiösen Patient:innen [6]. Dies ist relevant, da Spiritual Care für Gesundheitspersonal vor dem Hintergrund sinkender konfessioneller Bindung der deutschen Bevölkerung zunehmend an Bedeutung gewinnt. In einer Erhebung der evangelischen Kirche aus dem Untersuchungszeitraum 2022 bezeichneten sich 43 % der befragten Bevölkerung als konfessionslos, 25 % als katholisch, 23 % als evangelisch und 5 % als nichtchristlich religiös. Dabei gaben 48,5 % an, dass Religion „beim Umgang mit schwierigen Situationen“ gar keine Bedeutung habe [31]. In unserer Studie mit Gesundheitspersonal der Notfall- und Intensivversorgung gaben 28 % der Befragten an, keiner Religion zugehörig zu sein. Außerdem bezeichneten sich 53 % als nicht oder eher nicht gläubig, 57 % bezeichneten sich außerdem nicht als spirituell. Die Validierungsstudie des SCCQ hatte nur 9 % konfessionslose Befragte eingeschlossen [13]. In der Befragung beim Gesundheitspersonal in der Psychiatrie und Psychotherapie zeigten sich im Hinblick auf fehlende Religionszugehörigkeit und Gläubigkeit (33 % bzw. ca. 50 %) vergleichbare Zahlen zu unserer Erhebung [14]. In der Erfassung spiritueller Bedürfnisse spielen neben Religionszugehörigkeit auch kulturelle Unterschiede eine wichtige Rolle, da sie Einfluss auf die Betreuung am Lebensende haben können [10].

Für erfolgreiche spirituelle Kompetenz ist neben einer thematischen Sensibilisierung auch eine entsprechende Schulung erforderlich, besonders in den wiederholt gering eingeschätzten Kompetenzen wie der Dokumentationskompetenz. Dies geschieht bereits in verschiedenen Schulungsprogrammen. Im Rahmen des SpECi-Projekts werden Effekte einer curricularen Weiterbildung auf die Qualität der spirituellen Begleitung alter und/oder schwerstkranker Menschen untersucht [8]. Hier konnten präliminäre Ergebnisse zeigen, dass die Weiterbildung im Rahmen des SpECI-Projekts geeignet ist, Kompetenzen von Mitarbeitenden in der stationären Altenhilfe, Palliativstationen oder Hospizen zu steigern. In der BY.PASS-Studie konnten Patient:innen vor elektiven koronaren Bypassoperationen mit anschließenden Intensivaufenthalten nach persönlicher Präferenz eine psychologische oder spirituelle Intervention wählen. Die Endpunkte Krankenhausmorbidität und frühe Mortalität haben sich nicht zwischen den Interventionsgruppen und der Kontrollgruppe unterschieden, jedoch zeigte sich in beiden Interventionen eine Reduktion negativer Stimmung [25]. Weitere Studien zum Einfluss spiritueller Kompetenzen des Gesundheitspersonals und von Spiritual Care in der Intensiv- und Notfallversorgung sind dringend nötig, um Schulungen und die dafür erforderlichen Ressourcen vor dem Hintergrund zunehmender finanzieller und personeller Knappheit im Gesundheitswesen zu ermöglichen.

Diese Arbeit weist verschiedene Limitationen auf: Insgesamt wurde lediglich die Selbsteinschätzung von Gesundheitspersonal gemessen. Unklar bleibt, welche Auswirkungen die selbst eingeschätzte SCC und die damit verbundene Spiritual Care auf Patient:innenebene hat. Darüber hinaus kann keine Aussage über tatsächlich vorliegende oder ggf. fehlende SCC gemacht werden. Hier sind weiterführende Studien nötig, um das Erleben von Patient:innen und Zugehörigen zu untersuchen. Auch fehlt eine Untersuchung über ein allgemeingültiges Verständnis z. B. der zentralen Begriffe „Spiritualiät“ und „Gläubigkeit“. Hierzu hätte eine qualitative Befragung erfolgen müssen.

## Fazit für die Praxis

- Spiritual Care ist zunehmend Aufgabe des Gesundheitspersonals bei sinkender konfessioneller Bindung der Bevölkerung und kann durch Schulungen gesteigert werden.
- Unterschiede in spirituellen Kompetenzen zwischen den Geschlechtern und Professionen werden deutlich.
- Die eigene Spiritualität und Gläubigkeit der Studienteilnehmer:innen hat Auswirkungen auf ihre Spiritual-Care-Kompetenzen.
- Für eine wertschätzende Behandlung unter Einbeziehung von Spiritual Care müssen entsprechende Ressourcen vorhanden sein.

## Supplementary Information

Fragebogen mit Spiritual Care Competence Questionnaire (SCCQ) und demographischen Angaben

## Data Availability

Die in dieser Studie verwendeten Daten sind auf Anfrage und Nennung einer wissenschaftlichen Begründung beim korrespondierenden Autor erhältlich.
