# Supplementary material for: Spiritual care competences of healthcare workers in emergency and intensive care—a prospective questionnaire study
Source: Med Klin Intensivmed Notfmed. 2024 Oct 23;120(7):596–603. [Article in German] doi: 10.1007/s00063-024-01185-1 (PMC12504120; doi:10.1007/s00063-024-01185-1)
Supplement: Supplementary file 1 — Fragebogen mit Spiritual Care Competence Questionnaire (SCCQ) und demographischen Angaben [file 63_2024_1185_MOESM1_ESM.docx]

**Spirituelle Kompetenzen von Gesundheitspersonal in der Notfall- und Intensivversorgung – eine prospektive Fragebogenstudie**

**Spiritual Care Competence Questionnaire (SCCQ)**

**Wahrnehmungskompetenz:**

- Ich traue mir zu, spirituelle Bedürfnisse von Patienten wahrzunehmen.

Stimmt nicht ⃝ Stimmt kaum ⃝ Stimmt eher ⃝ Stimmt genau ⃝

- Ich traue mir zu, spirituelle Bedürfnisse von Angehörigen wahrzunehmen.

Stimmt nicht ⃝ Stimmt kaum ⃝ Stimmt eher ⃝ Stimmt genau ⃝

- Ich kann existentielle / spirituelle Bedürfnisse wahrnehmen, auch wenn die Patienten zur Religion wenig Bezug haben.

Stimmt nicht ⃝ Stimmt kaum ⃝ Stimmt eher ⃝ Stimmt genau ⃝

- Ich kann auch mit religionsfernen Patienten über ihre existentiellen / spirituellen Bedürfnisse reden.

Stimmt nicht ⃝ Stimmt kaum ⃝ Stimmt eher ⃝ Stimmt genau ⃝

- Ich bin in der Lage, Schmerzen / Leid von Patienten und ihren Angehörigen auszuhalten.

Stimmt nicht ⃝ Stimmt kaum ⃝ Stimmt eher ⃝ Stimmt genau ⃝

**Team-Spirit:**

- Wir sprechen regelmäßig im Team über die spirituellen Bedürfnisse der Patienten.

Stimmt nicht ⃝ Stimmt kaum ⃝ Stimmt eher ⃝ Stimmt genau ⃝

- In unserer Einrichtung (Praxis, Klinik usw.) besteht eine große Offenheit für das Themenfeld Spiritualität.

Stimmt nicht ⃝ Stimmt kaum ⃝ Stimmt eher ⃝ Stimmt genau ⃝

- Im Team tauschen wir uns regelmäßig über das Thema Spiritualität in der Patientenbegleitung aus.

Stimmt nicht ⃝ Stimmt kaum ⃝ Stimmt eher ⃝ Stimmt genau ⃝

- Im Team tauschen wir uns regelmäßig über unsere eigene Spiritualität aus.

Stimmt nicht ⃝ Stimmt kaum ⃝ Stimmt eher ⃝ Stimmt genau ⃝

- Im Team haben wir Rituale (z.B. Abschieds- und Unterbrechungsrituale), um gemeinsam mit probelmatischen Situationen umzugehen.

Stimmt nicht ⃝ Stimmt kaum ⃝ Stimmt eher ⃝ Stimmt genau ⃝

**Dokumentationskompetenz:**

- Ich kenne Instrumentarien (z.B. Themenliste) zur Erhebung einer spirituellen Kurz-Anamnese.

Stimmt nicht ⃝ Stimmt kaum ⃝ Stimmt eher ⃝ Stimmt genau ⃝

- Ich kenne Instrumente / Fragebögen zur strukturierten Erfassung spiritueller Bedürfnisse.

Stimmt nicht ⃝ Stimmt kaum ⃝ Stimmt eher ⃝ Stimmt genau ⃝

- Ich weiß, wie ich die spirituelle Anamnese meiner Patienten gut und nachvollziehbar dokumentieren kann.

Stimmt nicht ⃝ Stimmt kaum ⃝ Stimmt eher ⃝ Stimmt genau ⃝

**Selbsterfahrung und proaktive Öffnung:**

- Meine eigene Spiritualität prägt meinen Umgang mit anderen/kranken Menschen.

Stimmt nicht ⃝ Stimmt kaum ⃝ Stimmt eher ⃝ Stimmt genau ⃝

- Ich gehe regelmäßig auf Patienten zu, um deren spirituelle Bedürfnisse anzusprechen.

Stimmt nicht ⃝ Stimmt kaum ⃝ Stimmt eher ⃝ Stimmt genau ⃝

- Ich eröffne verbal, aber auch non-verbal einen "Raum", in dem der Patient ggf. spirituelle Anliegen einbringen kann, aber nicht gezwungen wird.

Stimmt nicht ⃝ Stimmt kaum ⃝ Stimmt eher ⃝ Stimmt genau ⃝

- Ich kümmere mich regelmäßig um die Vertiefung meiner eigenen Spiritualität (z.B. Besinnungstage, Meditation, Gottesdienstbesuch etc.).

Stimmt nicht ⃝ Stimmt kaum ⃝ Stimmt eher ⃝ Stimmt genau ⃝

- Ich besuche regelmäßig Fortbildungsveranstaltungen zu spirituellen Themen.

Stimmt nicht ⃝ Stimmt kaum ⃝ Stimmt eher ⃝ Stimmt genau ⃝

**Wissen über andere Religionen:**

- Ich weiß gut Bescheid darüber, welche religiösen Besonderheiten von Patienten aus anderen Religionsgemeinschaften berücksichtigt werden müssen.

Stimmt nicht ⃝ Stimmt kaum ⃝ Stimmt eher ⃝ Stimmt genau ⃝

- Ich achte darauf, dass die religiösen Besonderheiten von Patienten aus anderen Religionsgemeinschaften angemessen berücksichtigt werden.

Stimmt nicht ⃝ Stimmt kaum ⃝ Stimmt eher ⃝ Stimmt genau ⃝

**Gesprächsführungskompetenz:**

- Ich bin in der Lage, ein offenes Gespräch über existenzielle Themen zu führen.

Stimmt nicht ⃝ Stimmt kaum ⃝ Stimmt eher ⃝ Stimmt genau ⃝

- Ich bin in der Lage, ein offenes Gespräch über religiöse Themen zu führen.

Stimmt nicht ⃝ Stimmt kaum ⃝ Stimmt eher ⃝ Stimmt genau ⃝

**Proaktive Empowerment-Kompetenz:**

- Ich ermögliche meinen Patienten die Teilnahme an religiösen Handlungen / Feiern.

Stimmt nicht ⃝ Stimmt kaum ⃝ Stimmt eher ⃝ Stimmt genau ⃝

- Bei therapeutischen Entscheidungen achte ich auf religiöse / spirituelle Einstellungen, Haltungen und Überzeugungen des konkreten Patienten.

Stimmt nicht ⃝ Stimmt kaum ⃝ Stimmt eher ⃝ Stimmt genau ⃝

- Ich unterstütze meine Patienten dabei, ihre spirituellen Überzeugungen und Haltungen zu reflektieren.

Stimmt nicht ⃝ Stimmt kaum ⃝ Stimmt eher ⃝ Stimmt genau ⃝

- Ich achte auf den geeigneten Rahmen für spirituelle Gespräche.

Stimmt nicht ⃝ Stimmt kaum ⃝ Stimmt eher ⃝ Stimmt genau ⃝

**Demographische Angaben**

**Geschlecht:**

männlich ⃝ weiblich ⃝ divers ⃝

**Alter:**

18-29 ⃝ 30-39 ⃝ 40-49 ⃝ 50-59 ⃝ >60 ⃝

**Familienstand:**

verheiratet ⃝ mit Partner/-in zusammenlebend ⃝ geschieden ⃝ alleinstehend ⃝ verwitwet ⃝

**Profession:**

Arzt/Ärztin ⃝ PJ-Studiernde/r ⃝ Pflege ⃝ andere ⃝

**Fachbereich:**

Innere Medizin ⃝ Chirurgie/Orthopädie ⃝ Anästhesie ⃝ Neurologie ⃝ andere ⃝

**Bei aktueller Kursteilnahme von der Arbeitsgemeinschaft Intensivmedizin e.V. in Arnsberg, bitt Kurs angeben:**

Einführungskurs Intensivmedizin ⃝ Seminarkongress Notfallmedizin ⃝ keiner ⃝

**Berufstätig seit wie vielen Jahren?**

________

**Durchschnittliche Arbeitszeit in Stunden pro Woche?**

________

**Berufliche Zufriedenheit:**

Sehr zufrieden ⃝ zufrieden ⃝ es geht ⃝ unzufrieden ⃝ sehr unzufrieden ⃝

**Bezeichnen Sie sich selbst als spirituell?**

Ja ⃝ Nein ⃝

**Sind Sie ein (aktiv) gläubiger Mensch?**

Ja, unbedingt ⃝ ja, etwas ⃝ eher nein ⃝ nein, gar nicht ⃝

**Religionszugehörigkeit:**

Katholisch ⃝ Protestantisch ⃝ Muslimisch ⃝ Jüdisch ⃝ andere ⃝ keine ⃝
